# Understanding and preventing type 1 diabetes through the unique working model of TrialNet

**DOI:** 10.1007/s00125-017-4384-2

**Published:** 2017-08-02

**Authors:** Manuela Battaglia, Mark S. Anderson, Jane H. Buckner, Susan M. Geyer, Peter A. Gottlieb, Thomas W. H. Kay, Åke Lernmark, Sarah Muller, Alberto Pugliese, Bart O. Roep, Carla J. Greenbaum, Mark Peakman

**Affiliations:** 1 Diabetes Research Institute (DRI), IRCCS San Raffaele Scientific Institute, Via Olgettina 58, 20132 Milan, Italy; 2 Diabetes Center, University of California, San Francisco, CA USA; 3 Translational Research Program, Benaroya Research Institute, Seattle, WA USA; 4 Health Informatics Institute, University of South Florida, Tampa, FL USA; 5 Barbara Davis Center for Childhood Diabetes, University of Colorado School of Medicine, Aurora, CO USA; 6 St Vincent’s Institute, Fitzroy, VIC Australia; 7 Department of Medicine, St Vincent’s Hospital, University of Melbourne, Fitzroy, VIC Australia; 8 Lund University/CRC, Department of Clinical Sciences, Skane University Hospital, Malmö, Sweden; 9 Diabetes Research Institute, Department of Medicine, Division of Diabetes Endocrinology and Metabolism, Department of Microbiology and Immunology, Leonard Miller School of Medicine University of Miami, Miami, FL USA; 10 Department of Diabetes Immunology, Diabetes & Metabolism Research Institute, Beckman Research Institute at the City of Hope, Duarte, CA USA; 11 Department of Immunohaematology & Blood Transfusion, Leiden University Medical Center, Leiden, the Netherlands; 12 Diabetes Program Benaroya Research Institute, Seattle, WA USA; 13 Department of Immunobiology, Faculty of Life Sciences & Medicine, King’s College London, London, SE1 9RT UK; 14 National Institute for Health Research Biomedical Research Centre at Guy’s and St Thomas’ Hospital Foundation Trust and King’s College London, London, UK; 15 Institute of Diabetes, Endocrinology and Obesity, King’s Health Partners, London, UK

**Keywords:** Autoimmunity, Mechanistic studies, Review, Type 1 diabetes

## Abstract

Type 1 diabetes is an autoimmune disease arising from the destruction of pancreatic insulin-producing beta cells. The disease represents a continuum, progressing sequentially at variable rates through identifiable stages prior to the onset of symptoms, through diagnosis and into the critical periods that follow, culminating in a variable depth of beta cell depletion. The ability to identify the very earliest of these presymptomatic stages has provided a setting in which prevention strategies can be trialled, as well as furnishing an unprecedented opportunity to study disease evolution, including intrinsic and extrinsic initiators and drivers. This niche opportunity is occupied by Type 1 Diabetes TrialNet, an international consortium of clinical trial centres that leads the field in intervention and prevention studies, accompanied by deep longitudinal bio-sampling. In this review, we focus on discoveries arising from this unique bioresource, comprising more than 70,000 samples, and outline the processes and science that have led to new biomarkers and mechanistic insights, as well as identifying new challenges and opportunities. We conclude that via integration of clinical trials and mechanistic studies, drawing in clinicians and scientists and developing partnership with industry, TrialNet embodies an enviable and unique working model for understanding a disease that to date has no cure and for designing new therapeutic approaches.

**Electronic supplementary material:**

The online version of this article (doi:10.1007/s00125-017-4384-2) contains a slideset of the figures to download, which is available to authorised users.

## Introduction

Type 1 diabetes, a complex, three-stage, chronic autoimmune disease, accounts for 5–10% of all cases of diabetes. It is estimated that, worldwide, ~78,000 individuals under age 20 years are diagnosed with the disease annually, as well as a comparable number of adults. This disease rate has doubled every two decades since the middle of the last century and shows little sign of abating [1]. In 1974, this form of diabetes was first linked to an autoimmune process by the detection of circulating islet cell-specific autoantibodies [2]. Since then, global research efforts have transformed our understanding of the disease through classical studies of epidemiology, pathology, genetics and natural history. As a result, we now know that type 1 diabetes is an autoimmune disease arising from the destruction of pancreatic insulin-producing beta cells [3]. But behind that simple statement lie many complexities. Although strong links to HLA class I and II genes indicate a role for T lymphocytes in the disease process, numerous other cellular and molecular immune pathways have been implicated and disease initiators and drivers remain to be identified with certainty.

**Fig. 1:**
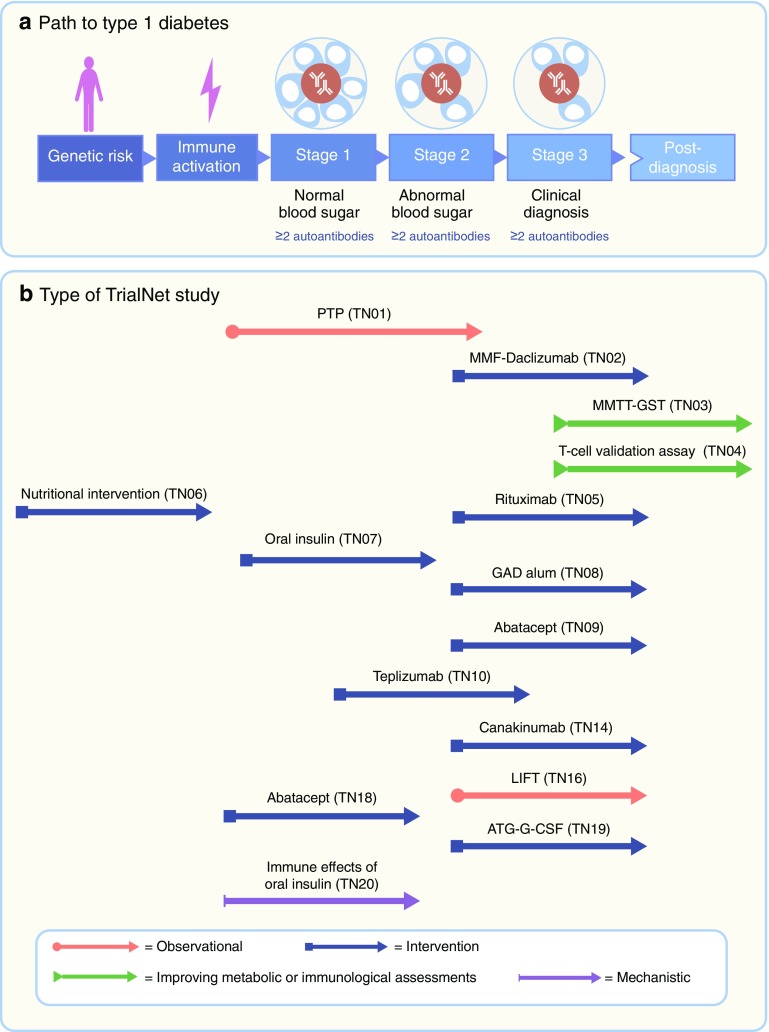
Type 1 diabetes: a disease with three stages of pathology and various opportunities for intervention with disease-modifying therapies and mechanistic analysis. (**a**) Stage 1 is the start of the disease—there are no symptoms and blood sugar remains normal but the autoimmune process has already begun, manifested by multiple autoantibodies against beta cell targets. In stage 2, like stage 1, autoimmunity is the key feature and there are no symptoms; however, blood sugar control has now become abnormal due to loss of beta cells. From stages 1 and 2, there is an extremely high risk of progression to stage 3, when symptoms of diabetes emerge (thirst, weight loss and fatigue) due to significant beta cell loss, and the clinical diagnosis is made. (**b**) Each stage of the disease is encompassed within TrialNet and offers an opportunity for interventions or mechanistic analysis. Examples of TrialNet studies (concluded and ongoing) are listed in association with the targeted disease stage. ATG-G-CSF, anti-thymocyte globulin+granulocyte colony stimulating factor; GST, glucagon stimulation test; LIFT, long-term investigative follow-up; MMF, mycophenolate mofetil; MMTT, mixed meal tolerance test; PTP, Pathway to Prevention study. Abatacept is a CTLA-4–immunoglobulin; canakinumab is an anti-IL1β monoclonal antibody (mAb); rituximab is an anti-CD20 mAb; teplizumab is an anti-CD3 mAb

The 1974 discovery did not just achieve the subdivision of a syndrome—it is also of great practical importance. Autoantibodies directed against specific targets in the beta cell show high sensitivity and specificity for the identification of individuals at risk of future development of type 1 diabetes, in the absence of blood glucose abnormalities [4]. The possibility of studying a disease prodrome sets type 1 diabetes apart from almost all other immune-mediated inflammatory diseases and has additional ramifications. Recently, for example, it has spurred proposals for a new type 1 diabetes nosology, in which stage 1 disease is defined as the simple presence of autoantibodies; in stage 2 this is accompanied by abnormal blood glucose control and stage 3 is frank diabetes [5] (Fig. 1a). But perhaps of greatest importance, the ability to identify at-risk individuals in stages 1 and 2 has provided a setting in which type 1 diabetes prevention can be contemplated, as well as an unprecedented opportunity to study the evolution of the disease.

Against this backdrop, Type 1 Diabetes TrialNet was formed in 2003, funded by the National Institute of Diabetes and Digestive and Kidney Disease (NIDDK) of the National Institutes of Health and the JDRF, with the objective of developing a clinical network approach to type 1 diabetes prevention, while at the same time promoting studies of disease pathogenesis [6]. In this review, we will focus on the latter function.

## Type 1 Diabetes TrialNet

Type 1 Diabetes TrialNet (TrialNet) has developed into an international network of researchers who are exploring ways to prevent, delay and reverse the progression of type 1 diabetes. TrialNet was established in response to the Surgeon General’s Report ‘Healthy People 2000’. This report identified diabetes as a US national health objective and in response the US Congress created the Diabetes Research Working Group (DRWG). One of this group’s earliest recommendations was the setting up of a programme of research studies and clinical trials to prevent type 1 diabetes [6].

Since 2016, TrialNet has conducted clinical trials with researchers from 18 clinical centres in the USA, Canada, Finland, UK, Italy, Germany, Sweden, Australia and New Zealand. In addition, more than 150 medical centres and physician offices are participating in the network, offering major research opportunities (www.trialnet.org/our-research). One of the lynchpin programmes is the Pathway to Prevention study (protocol TN01), through which relatives of individuals with type 1 diabetes are screened for the presence of circulating autoantibodies. Those identified as being at increased risk (individuals in stage 1 or 2 of the disease) are followed up and offered clinical trial opportunities. During follow-up, individuals attend regular assessment visits that include the collection of blood for continued evaluation of islet cell autoantibody status and the collection of longitudinal samples for mechanistic studies, as well as determination of metabolic status (dysglycaemia, frank diabetes) and systematic collection of metadata (Table 1). In addition to TN01, TrialNet has conducted pivotal clinical trials with the following aims: (1) to determine whether new treatments can delay or prevent the onset of clinically overt type 1 diabetes in individuals at prediabetic stages and (2) to preserve insulin production in individuals newly diagnosed with clinical type 1 diabetes (Fig. 1b). The collection of an array of longitudinal bio-samples from these studies (both concluded and ongoing) provides the added value of potential for understanding mechanisms of disease and therapeutic effects, as well as identifying appropriate biomarkers.

**Table 1:** Pathway to Prevention study (TN01)

Serological status	Screened^a^	Enrolled in monitoring^b^
All individuals	178,648	5506
Autoantibody negative^c^	170,487	155
Positive for one autoantibody^c^	3273	2144
Positive for two or more autoantibodies^c^	4888	3207

Data as of 31 December 2016; a total of 835 individuals progressed to type 1 diabetes

^a^Eligible individuals who were genetically at risk and who were tested for autoantibodies through TrialNet

^b^Individuals who were eligible for and enrolled in active monitoring through TrialNet for metabolic and clinical markers on an annual or semi-annual basis

^c^Autoantibody-negative individuals underwent annual monitoring and multiple autoantibody-positive individuals underwent semi-annual monitoring. Most individuals confirmed positive for a single autoantibody underwent annual monitoring, although some converted to semi-annual monitoring

It is worth noting that TN01 is not a ‘traditional’ natural history study but rather a ‘feeder’ study designed to identify individuals at various disease stages for appropriate prevention trials and to help to understand disease progression. As a result, there is scant information on the age of seroconversion of TrialNet participants, or on the types of autoantibodies with which they first present. This is a potentially important limitation, since data have emerged to indicate that different disease phenotypes may exist (e.g. *HLA-DR4/DQ8* haplotype is linked to insulin autoantibodies at seroconversion, while *HLA-DR3/DQ2* haplotype is linked to anti-GAD at seroconversion [7]). These phenotypes may reflect different endotypes (i.e. different aetiopathogenesis) and/or may be suited to different prevention strategies and TrialNet can begin to address these issues in future clinical trials, either through using HLA genotype as a surrogate, or through modification of participant screening strategies.

## TrialNet ancillary studies

Since inception, TrialNet has thus assembled an enviable bioresource of longitudinal samples and metadata, which is open to access by the global research community through an ancillary studies programme. The collection contains over 70,000 samples gathered from over 7500 donors at various follow-up time points and includes genomic DNA, whole-blood RNA, serum, plasma and peripheral blood mononuclear cells. Further, specialised sampling and additional metadata can also be acquired in real-time studies. This is the ‘living biobank’ approach, which provides investigators with the opportunity to obtain on-demand biological samples from selected individuals who are engaged in TrialNet protocols. Studying these samples offers opportunities for natural history studies and can provide critical understanding as to how or why specific intervention strategies did or did not deliver a specified primary study outcome.

**Fig. 2:**
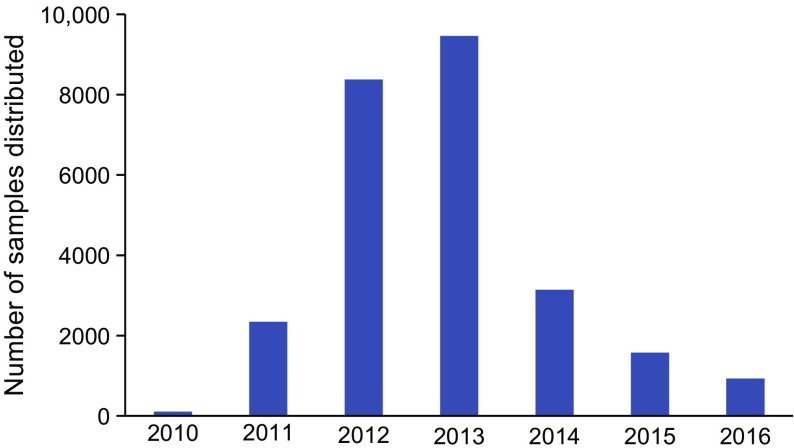
Archived TrialNet samples distributed over the years. The numbers of TrialNet archived samples allocated to perform ancillary studies are shown according to year of distribution

As of 31 December 2016, more than 25,500 archived samples have been distributed to 35 laboratories for 48 ancillary studies (Fig. 2).

**Fig. 3:**
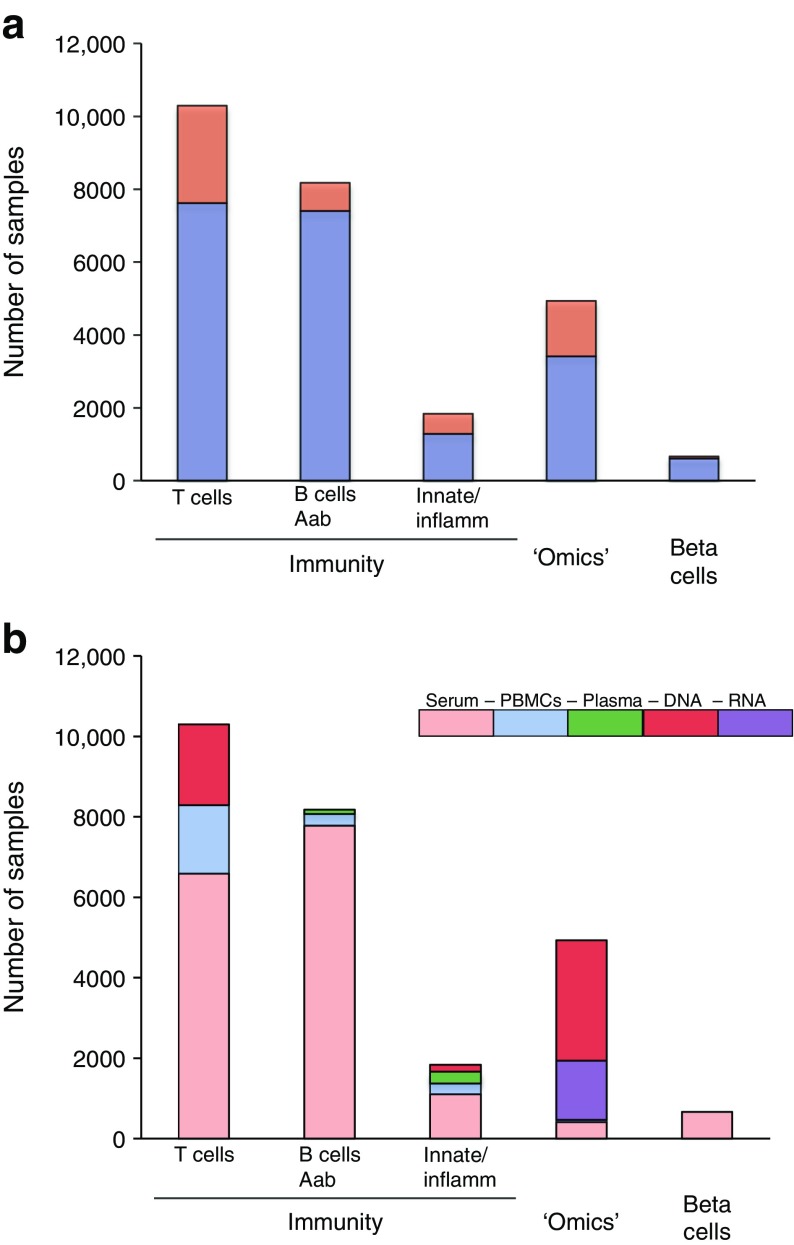
Archived TrialNet samples distributed to perform ancillary studies. (**a**) The number of TrialNet archived samples distributed to perform ancillary studies are shown according to the focus of the ancillary study (immunity [further subdivided into those focused on T cells, B cells and autoantibodies (Aab), and innate immunity and inflammation (innate/inflamm)], ‘omics’ and beta cells). TrialNet studies from which samples were collected are indicated (blue bars, TN01, Pathway to Prevention study; orange bars, all other TrialNet studies). (**b**) The number of TrialNet archived samples distributed to perform ancillary studies are shown according to the focus of the ancillary study (immunity [further subdivided into those focused on T cells, B cells and Aab, and innate/inflamm], ‘omics’ and beta cells) and by type of sample distributed (serum; peripheral blood mononuclear cells [PBMCs], plasma, DNA and RNA)

Thus far, questions addressed in TrialNet ancillary studies have focused on the immune system (mainly studies on T cells) but there are also studies on beta cells as well as studies utilising multi-dimensional platform technologies (‘omics’) (Fig. 3a). Serum and plasma represent the most-requested types of samples across all types of studies (Fig. 3b).

## TrialNet ancillary studies signpost key breakthroughs

**Fig. 4:**
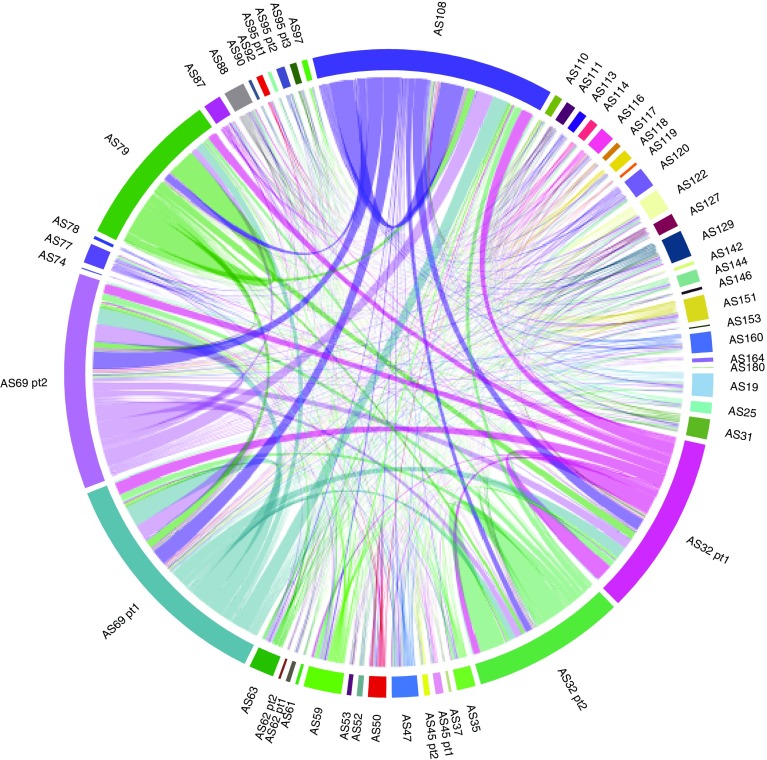
Ancillary studies performed on the same TrialNet donors. Chord diagram of the components (TN ancillary studies, designated AS) and their interactions (connecting lines). Studies that analysed the same TrialNet donors are connected by lines: the wider the line, the higher the number of individuals common to the different ancillary studies. The scale and width of the TrialNet ancillary study component reflects the number of donors shared across other TrialNet ancillary studies

The TrialNet programme of ancillary studies has provided more than 40 investigators with more than 25,500 samples and as such there is a wealth of information from work already done addressing important questions. Of relevance is the presence of more than 2900 donors who were analysed by different TrialNet investigators to address various scientific questions (Fig. 4). This provides a great opportunity to integrate data from different studies performed in various laboratories on the same individuals. To best enhance this asset, TrialNet is currently seeking new collaborations that aim to analyse and integrate data obtained from existing ancillary studies. This will provide added value to TrialNet, its collaborators and the type 1 diabetes research community. In addition to this work in progress, several ancillary studies have been completed to date and have provided transformational insights, changing the way in which we view the disease. A selection of these and their key breakthroughs are described briefly below.

### Definition of type 1 diabetes endotypes

From a clinical standpoint, it is obvious that type 1 diabetes is a heterogeneous disease. For example, it can manifest very aggressively in a 3-year-old child who has rapid symptomatic onset, type-1-diabetes-associated autoantibodies, exceedingly low C-peptide levels, disease-predisposing HLA genes and an associated inflammatory process such as coeliac disease. But type 1 diabetes can also occur in a 40-year-old individual with limited HLA risk, detectable C-peptide, a mild, indolent onset and no other autoimmune diseases. With insulin replacement by injection being the only approved therapy for type 1 diabetes, there has seemed little need to identify disease heterogeneity from a clinical–pathological perspective [8]. However, this heterogeneity is emerging as a potentially important concept. Data from the T1D Exchange Clinic Network, for instance, demonstrated heterogeneity in loss of insulin secretion at each stage of disease [9]. Other studies, especially those of pancreas samples post-mortem in the Network for Pancreatic Donor (nPOD) collection, revealed considerable variability in the degree of residual insulin-reactive staining in recent-onset disease [10]. This prompted a more focused set of questions around whether endotypes exist within type 1 diabetes, which could have dramatic impact on our understanding of disease processes and therapeutic approaches. An endotype is a disease subtype defined by a distinct functional or pathobiological mechanism [11]. Results generated in a TrialNet ancillary study took the first steps towards the description of type 1 diabetes endotypes, using two complementary analyses of tissue and blood to identify disease-related subphenotypes in individuals with type 1 diabetes. The blood-based study (including samples from TN01) showed that approximately one-half of the individuals, analysed close to diagnosis, had autoreactive T cell responses characterised by the proinflammatory cytokine IFN-γ, whereas one-half were distinguished as having IL-10 responses (typical of immune regulation) along with significantly fewer autoantibodies. In the tissue-based study, analysis of pancreases obtained close to disease diagnosis (through the nPOD network) showed that approximately one-half of the individuals had an inflammatory islet infiltration distinguished by high numbers of CD20^+^ B lymphocytes, whereas one-half had sparse infiltration and significantly fewer B lymphocytes [12]. Thus, this work shows the existence of blood and tissue phenotypes (inflammatory CD20^High^ vs regulatory CD20^Low^) that are likely to be related to each other and to variable rates of disease progression. Despite the fact that formal proof of such a link is still lacking–since blood and pancreas samples were collected from distinct individuals–this study provides the first example of a pathophysiological basis for disease heterogeneity that may have direct relevance to stratification for therapeutic trials [12].

### Discovery of circulating markers of beta cell killing and/or stress

Another important gap in type 1 diabetes research remains the lack of biomarkers that reflect an authentic signature of beta cell death and/or stress. Resolving this issue may also improve our ability to optimally time immunomodulatory treatments and other forms of interventions in type 1 diabetes. Several investigators have recently developed methods for assessing beta cell death in vivo by measuring the relative amount of beta cell-derived insulin encoding DNA (*INS* DNA) in the circulation. This analysis involves quantitative PCR amplification of *INS* DNA, with epigenetic marks that identify the DNA source as cells that actively transcribe insulin. Since the only significant source of non-methylated *INS* DNA in the body is the beta cell, the level of unmethylated *INS* DNA in the circulation reflects the active rate of beta cell death [13]. A recent TrialNet ancillary study determined levels of beta cell killing using this approach in individuals at risk of type 1 diabetes (from TN01). This study demonstrated that beta cell death can be found before the onset of type 1 diabetes. Importantly, the study gave insight into the tempo of the disease: while episodes of beta cell killing associated with a decline in insulin secretion were detected in the prediabetes period, there was a dramatic increase in killing in the peri-diagnosis period [14]. Although the ‘unmethylated *INS* DNA’ assay has yet to be fully validated [15], these data support the notion that a blood test that measures beta cell death in the setting of type-1-diabetes-associated autoimmunity can potentially be used in the future as the first surrogate marker of its kind, able to monitor beta cell ‘health-status’ during prevention and intervention studies.

Beta cells, like all professional secretory cells, naturally undergo high levels of endoplasmic reticulum stress as a result of their normal secretory physiology [16]. A hallmark of beta cell endoplasmic reticulum dysfunction is the accumulation and secretion of inadequately processed proinsulin molecules [17]. Therefore, using the proinsulin/C-peptide ratio (PI/C) as a non-invasive marker of beta cell dysfunction could provide a means to identify individuals at risk of developing type 1 diabetes prior to the onset of massive beta cell destruction. Sims et al recently reported that in archived samples from autoantibody-positive individuals enrolled in the Pathway to Prevention study (TN01), fasting PI/C was significantly increased ∼12 months prior to onset of type 1 diabetes [18]. Elevations were most pronounced in children aged ≤10 years, whose median PI/C was threefold higher than that of individuals who did not progress to type 1 diabetes. However, even among the entire group, increased PI/C was associated with increased odds of progression to type 1 diabetes after correction for age and BMI [18]. PI/C remains likely to be an informative and useful component of type 1 diabetes risk prediction algorithms, as well as an important biomarker of beta cell dysfunction in treatment trials. In addition, these results were confirmed by recent data demonstrating that in situ (in pancreas sections from diabetes autoantibody-positive donors), insulin area and beta cell mass can be maintained prior to disease onset and that production of proinsulin increases [19]. Using high-resolution confocal microscopy, this study revealed a high accumulation of vesicles containing proinsulin in beta cells from autoantibody-positive donors. This suggests either a defect in proinsulin conversion or an accumulation of immature vesicles due to an increase in insulin demand and/or to a dysfunction in vesicular trafficking. Taken together, these data suggest that prevention during stage 1 of the disease, when beta cell mass is still intact, could be a successful therapeutic strategy.

### Discovery of circulating microRNAs associated with disease progression

It is now clear that rates of progression to clinical symptoms vary significantly among individuals [5]. Thus, it would be desirable to identify additional biomarkers that could improve prediction beyond that afforded by autoantibody positivity. A recent study provided initial evidence that levels of selected circulating microRNAs are associated with increased risk of disease progression [20]. Augmented levels of these microRNAs conferred increased risk in young autoantibody-positive individuals enrolled in the Pathway to Prevention study (TN01). Moreover, the levels of several of the microRNAs associated with disease progression correlated with multiple OGTT indices of beta cell function, including both glucose and C-peptide area under the curve, peak C-peptide, and composite metabolic scores. Of note, several of these microRNAs have known influence on beta cell function and insulin secretion. Additional studies are needed to validate and expand these findings, which can be correlated with immunological and beta cell stress/death markers.

### Discovery of an immunological biomarker of C-peptide level decline

TrialNet performed a clinical trial of immunological co-stimulation blockade using CTLA-4–immunoglobulin (abatacept) in individuals newly diagnosed with type 1 diabetes (TN09). Two years of continuous treatment was associated with a slower decline of beta cell function and the beneficial effect was sustained for at least 1 year after therapy cessation [21]. To better understand the mechanism of this therapeutic effect, Orban and colleagues conducted an ancillary study to analyse immune cell subsets in treated and control individuals, using polychromatic flow cytometry [22]. The study revealed that continuous co-stimulation blockade is associated with an inversion of conventional naive/memory CD4^+^ T cell subset status, marked by a significant reduction in circulating CD4^+^CD45R0^+^CD62L^+^ central memory (CM) T cells and concomitant increase in CD4^+^CD45R0^−^CD62L^+^ naive T cells. These changes reverted after treatment cessation, supporting a direct drug effect. Importantly, the study also revealed that in placebo-treated individuals there was an increase in the number of circulating CM CD4^+^ T cells and a decrease in CD4-naive/CM T cell ratio, significantly associated with a subsequent rise in the rate of C-peptide decline. Abatacept treatment significantly altered this association. Mechanistically, this implies that the effect of co-stimulation blockade on naive/CM status of CD4^+^ T cells is linked to its therapeutic effect [22]. These data are the first to reveal an immunological biomarker of C-peptide level decline in individuals in whom type 1 diabetes has recently developed. This marker could assist in the prediction of progression of immunological damage to beta cells at various stages of the disease and provides a strong rationale for early therapeutic interference in co-stimulation and generation/maintenance of immune memory in type 1 diabetes. Currently, a TrialNet study of abatacept is being conducted in those at stage 2 of disease (i.e. with two autoantibodies and normal glucose tolerance) (www.trialnet.org/our-research/prevention-studies) (TN18, Fig. 1b).

### Novel insights into disease pathogenesis

Type 1 diabetes is typically described as an immune-mediated disease in which T cells drive the specific destruction of pancreatic insulin-producing beta cells. Recent data arising from TrialNet ancillary studies indicate that this T cell-centric view may need broadening. The role of B lymphocytes in type 1 diabetes is poorly understood despite autoantibodies being known robust predictive biomarkers. From results of studies making use of TrialNet samples, it is now becoming apparent that a breach in B lymphocyte tolerance is also a major contributor to type 1 diabetes. The trigger for these findings is the success of TN05, a clinical trial in individuals with new-onset type 1 diabetes, using monoclonal anti-CD20 antibody therapy (rituximab) to deplete CD20^+^ B cells. Those receiving active treatment showed reduced decline in C-peptide secretion in the year following introduction of therapy and better glycaemic control with reduced requirements for exogenous insulin [23]. Disappointingly, however, clinical efficacy was not sustained much beyond 1 year, prompting initiation of several TrialNet ancillary studies designed to better understand this phenomenon [24]. Meffre and co-workers, who previously reported defective central and peripheral B cell tolerance checkpoints resulting in the accumulation of self-reactive mature B cells in individuals with type 1 diabetes [25], investigated whether anti-B cell therapy modifies the frequency of autoreactive B cells. They found that B cell depletion did not change the frequencies of autoreactive and polyreactive B cells, which remained elevated in all individuals after rituximab treatment. There was a limited proliferative history of autoreactive B cells after treatment but these clones were newly generated B cells and not self-reactive B cells that had escaped depletion and repopulated the periphery through homeostatic expansion. Thus, anti-B cell therapy may provide a temporary reduction in autoimmune processes through B cell depletion. However, repletion with autoreactive B cells after this therapy may explain the relapse that occurs in many autoimmune individuals. This study adds weight to the emerging concept that autoreactive B cells have a major pathogenic role in human type 1 diabetes. Moreover, these and other studies support the idea of trialling sequential therapy: first depleting B cells and then addressing resurging T cell activation.

In accordance with this view, Cambier and colleagues demonstrated that high-affinity insulin-binding B cells are present in healthy individuals exclusively in the anergic naive compartment, while these insulin-binding B cells lose their anergic phenotype in individuals who are at risk of diabetes (TN01 participants) or who have new-onset (< 1 year) type 1 diabetes [26]. These findings suggest that disruption of B cell anergy may predispose individuals towards autoimmunity, consistent with a potential early role for B cells in the development of type 1 diabetes.

A further, often neglected, component of the immune responses in individuals with type 1 diabetes has been that of innate immune pathways. A careful examination of the peripheral blood of relatives of individuals with type 1 diabetes (TN01) and of individuals with established disease has revealed a reduced number of circulating neutrophils. Indeed, the higher the risk of progression to diabetes, the more pronounced the reduction. This observation was possible through direct access to fresh blood from donors participating in TN01 using the Living Biobank approach. The observed reduction in circulating neutrophils is accompanied by the novel finding that neutrophils infiltrate the pancreas of individuals with type 1 diabetes, studied as cadaveric organ donors, suggesting that a disturbance in peripheral neutrophil regulation arises in parallel with neutrophil pancreas marginalisation [27]. Reduced circulating neutrophils have also been found in individuals with type 1 diabetes (<100 days from diagnosis) enrolled in five different TrialNet intervention trials (TN02, TN05, TN08, TN09 and TN14; Fig. 1b) [28]. It remains to be defined whether this reduction may reflect the aetiopathology of disease or serve as a clinical biomarker of a subgroup of individuals who develop diabetes.

Additional evidence pointing to the presence of innate immune mechanisms during the early stage of the disease (stage 1) comes from Hessner’s group [29]. They used plasma-induced transcriptional changes to investigate immune states within type 1 diabetes families (TN01 donors) and how these alter over time with either progression or non-progression to diabetes. While the plasma of individuals with recent-onset diabetes induced a distinct signature relative to that of control non-diabetic individuals and those at low or high risk of developing type 1 diabetes (i.e. autoantibody-negative siblings who either lacked or possessed *HLA*-*DR3* and/or *-DR4* haplotypes, respectively), the signatures of these healthy cohorts were also distinct from one another [30]. Notably, the signature of low-risk individuals exhibited the most robust induction of innate inflammatory transcripts (e.g. IL-1B, chemokine [C-C motif] ligand 2 [CCL2], CCL3, CCL7, chemokine [C-X-C motif] ligand 1 [CXCL1], CXCL2, CXCL3, CD14 and triggering receptor expressed on myeloid cells 1 [TREM1]). Pathway analyses revealed a continuum of immune states across the four cohorts. The plasma of individuals with recent-onset type 1 diabetes induced signatures intermediate between those at low and high risk of developing the disease, such that inflammation decreased and regulation increased across the low risk → recent-onset disease → high risk → non-diabetic donor continuum [30]. These results not only shed some light on the importance of innate immunity pathways in the early stage of the disease but they may also present new therapeutic opportunities before autoantibody development.

## Maximising the potential of a networked bioresource in a complex disease setting

The studies outlined above clearly provide important breakthroughs but thus far ancillary studies have been predominantly single-centre, focused on one hypothesis/question and generated from samples obtained from a few selected donors. In the era of team science, ‘big data’ and systems biology approaches allied with high-throughput technology, TrialNet will best move forwards by fostering models for mechanistic studies that are bigger in scope and represent and integrate different investigators and the myriad of data opportunities. One approach that is gaining considerable attraction and will become a future focus is to assemble these technologies to address fundamentally important questions relevant to the TrialNet mission, such as the discovery of biomarkers and mechanisms of progression through the disease stages. TrialNet will be able to play a major role in coordinating external collaborations and data integration to achieve this goal but success will also depend upon engagement with the relevant experts. The revised model envisages mechanistic studies that will be considered an integral, core part of TrialNet activity and no longer ‘ancillary’.

Overall, our perspective on the achievements of the TrialNet model of conducting wide-ranging studies ancillary to its trials is that it has pushed the field forwards in multiple directions, and has begun to reveal biomarkers and deliver the mechanistic understanding required to combat this complex and heterogeneous disease. We anticipate that the findings expected to emerge soon from multi-dimensional analyses addressing key transition points in the disease will build on this platform, maximising the opportunities provided by this precious resource.

## Electronic supplementary material

ESM Downloadable slideset (PPTX 586 kb)

## Abbreviations

CM: Central memory
nPOD: Network for Pancreatic Donor
PI/C: Proinsulin/C-peptide ratio
