# Supplementary material for: Understanding and preventing type 1 diabetes through the unique working model of TrialNet
Source: Diabetologia. 2017 Aug 2;60(11):2139–47. doi: 10.1007/s00125-017-4384-2 (PMC5838353; doi:10.1007/s00125-017-4384-2)
Supplement: Supplementary file 1 — (PPTX 586 kb) [file 125_2017_4384_MOESM1_ESM.pptx]

## Slide 1
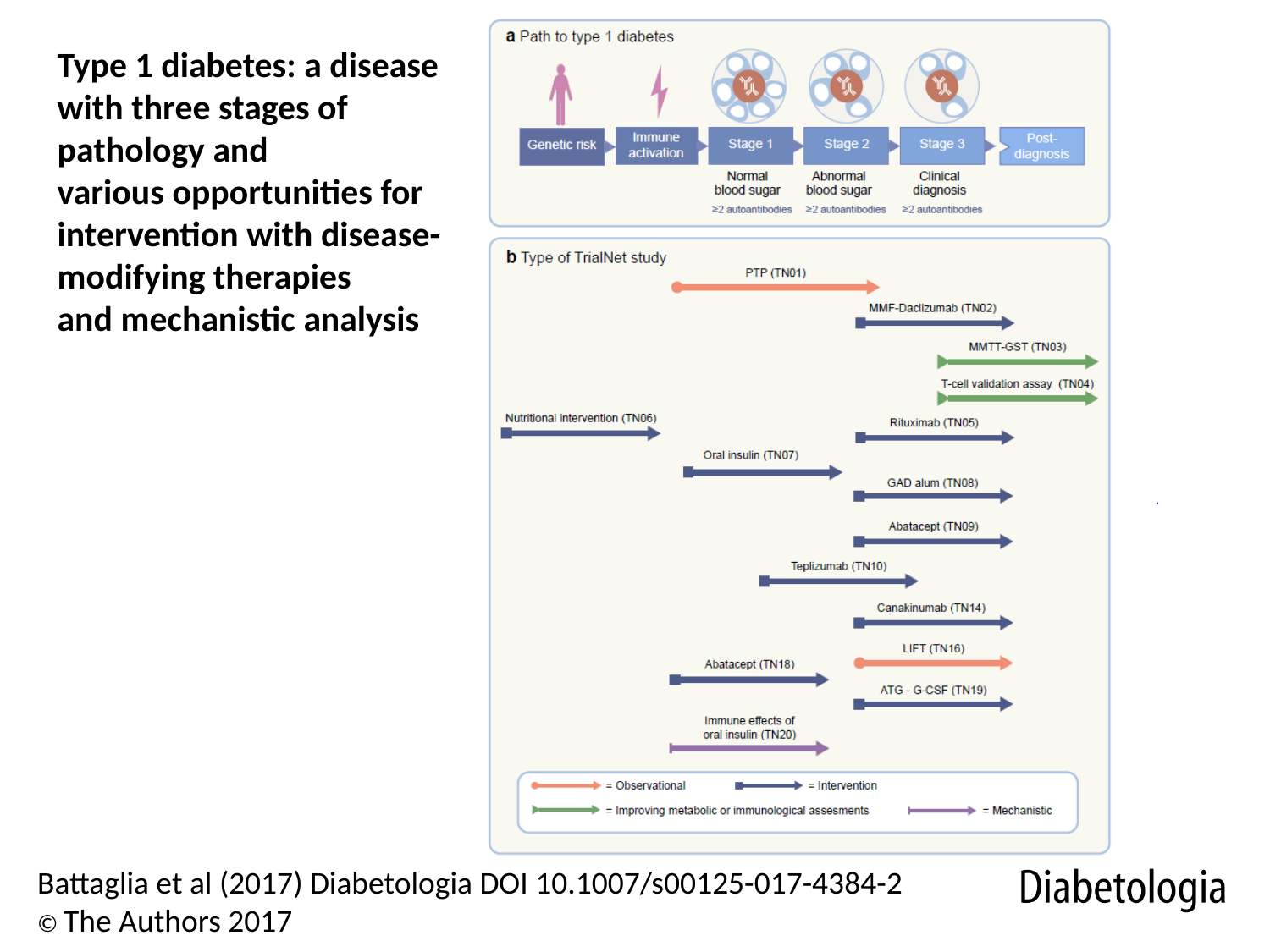

Type 1 diabetes: a disease with three stages of pathology and
various opportunities for intervention with disease-modifying therapies
and mechanistic analysis
Battaglia et al (2017) Diabetologia DOI 10.1007/s00125-017-4384-2
© The Authors 2017

## Slide 2
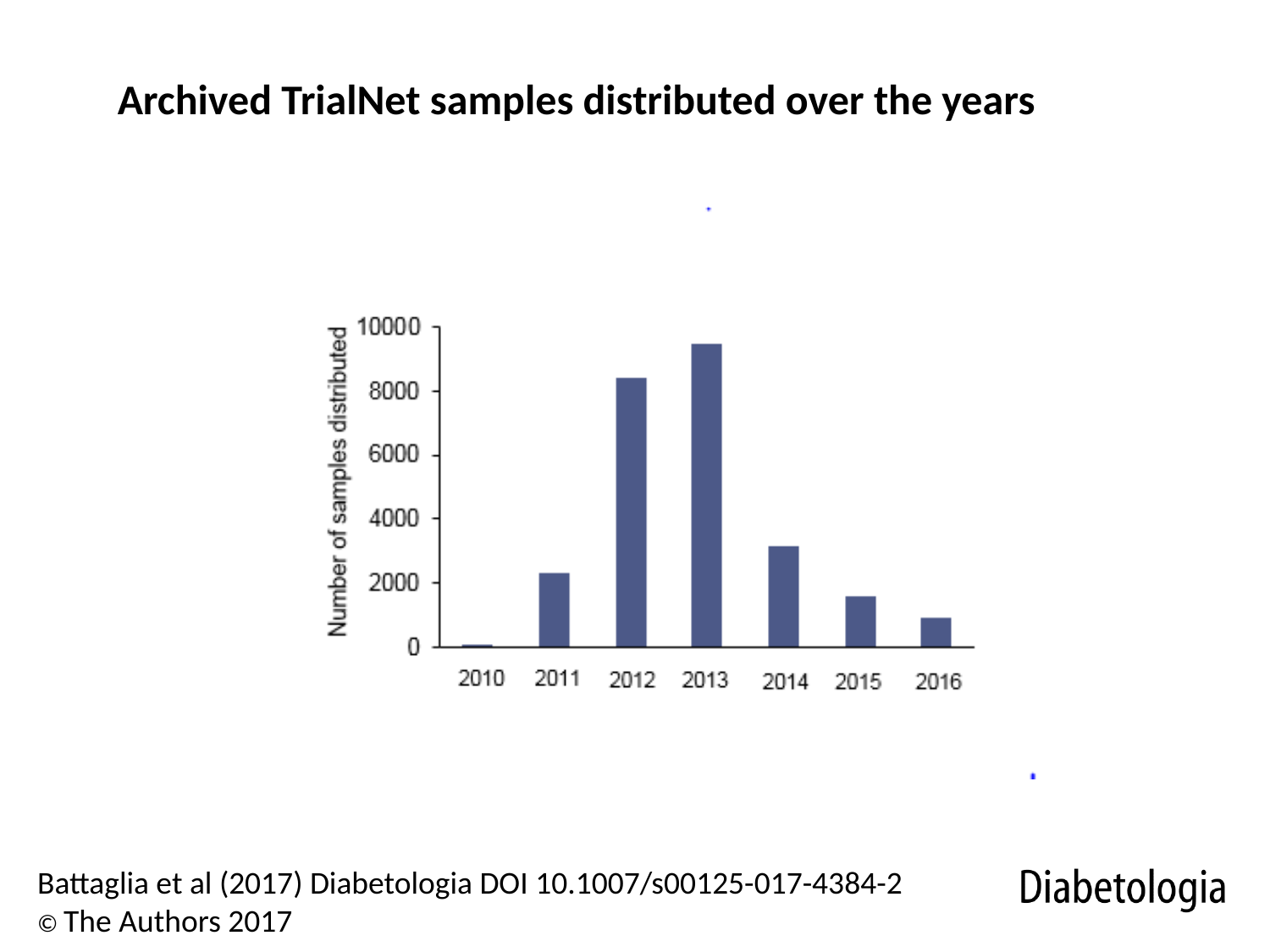

Archived TrialNet samples distributed over the years
Battaglia et al (2017) Diabetologia DOI 10.1007/s00125-017-4384-2
© The Authors 2017

## Slide 3
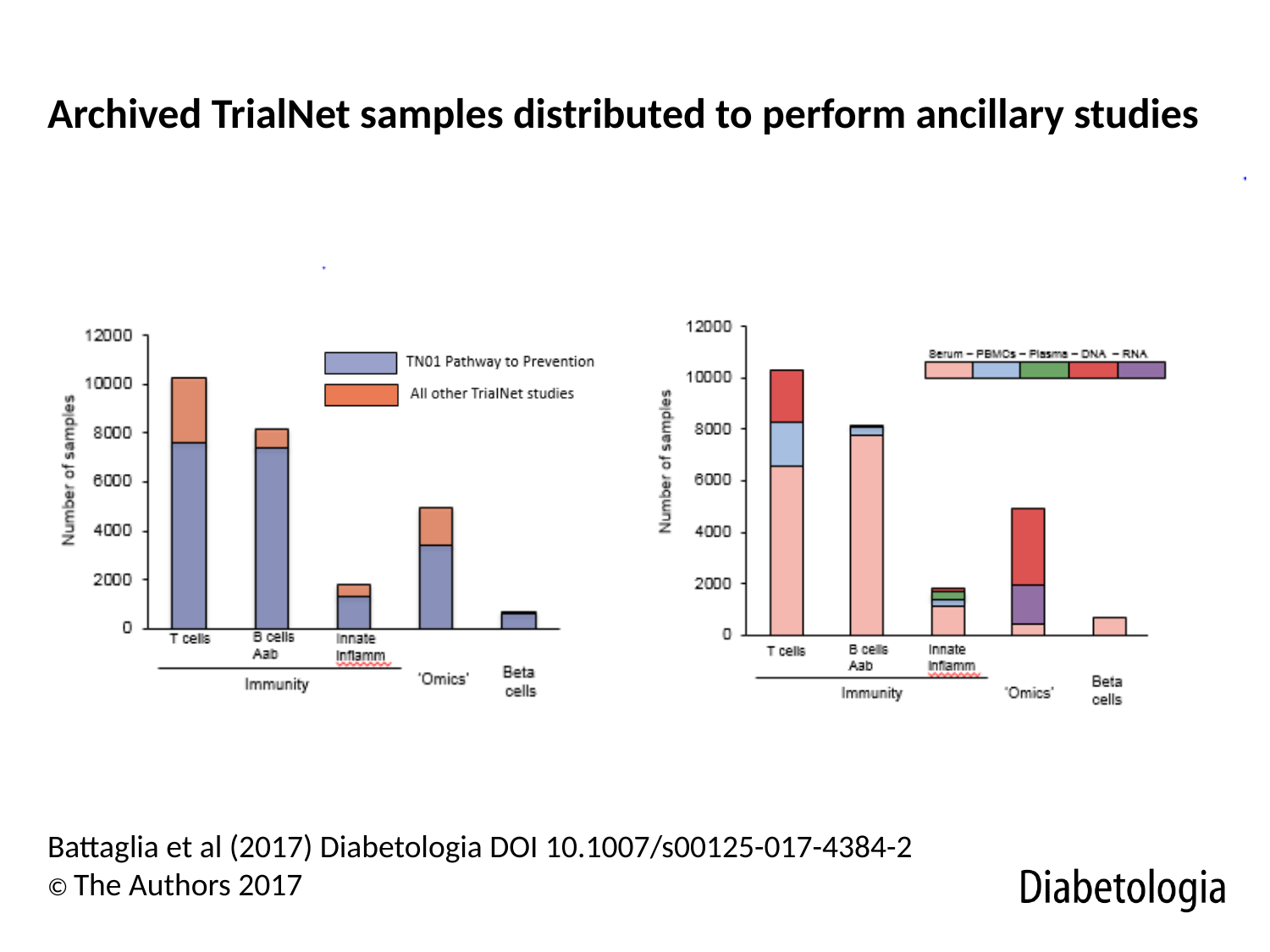

Archived TrialNet samples distributed to perform ancillary studies
Battaglia et al (2017) Diabetologia DOI 10.1007/s00125-017-4384-2
© The Authors 2017

## Slide 4
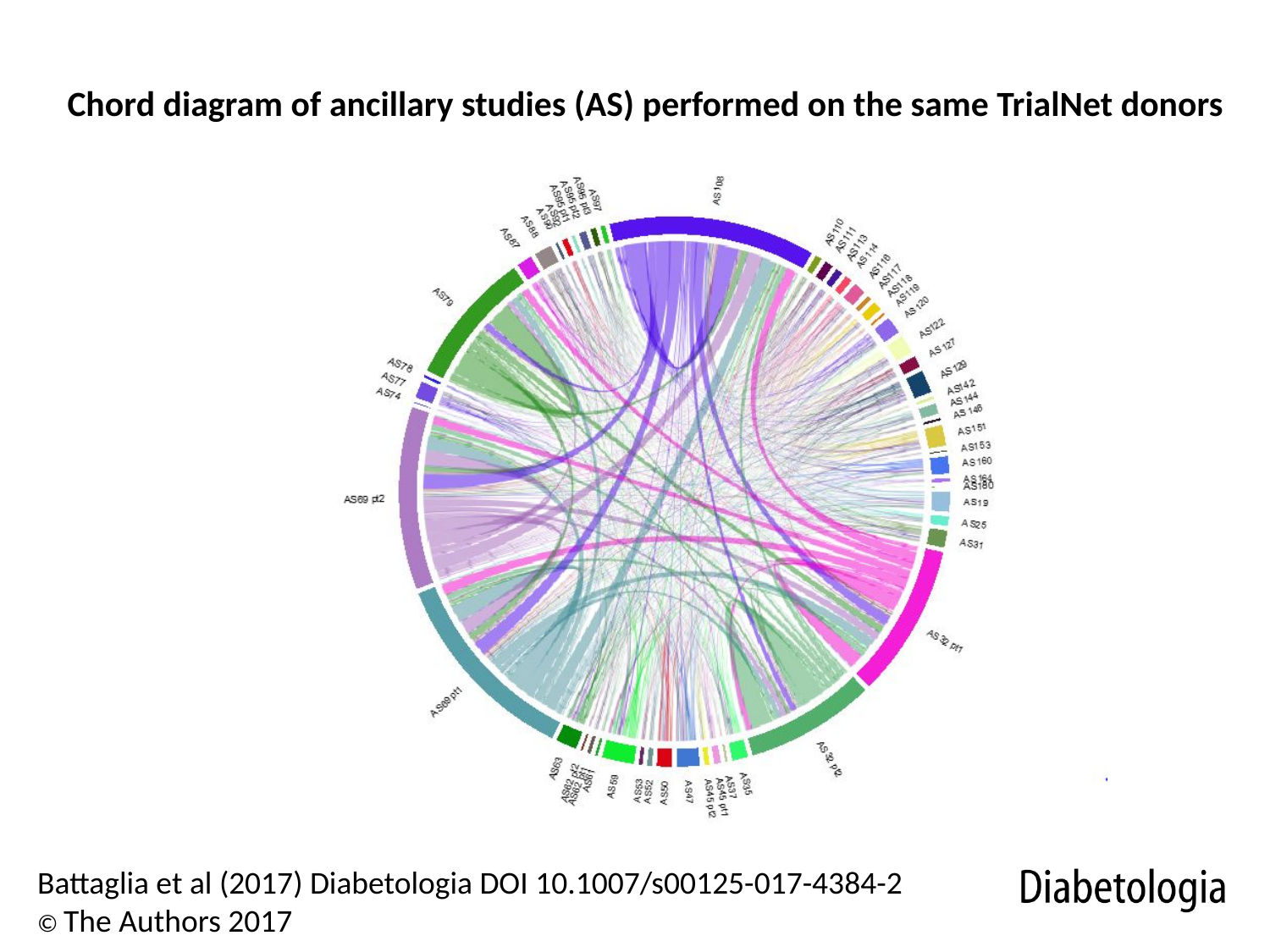

Chord diagram of ancillary studies (AS) performed on the same TrialNet donors
Battaglia et al (2017) Diabetologia DOI 10.1007/s00125-017-4384-2
© The Authors 2017
